# Potential impact of the implementation of multiple-criteria decision analysis (MCDA) on the Polish pricing and reimbursement process of orphan drugs

**DOI:** 10.1186/s13023-016-0388-0

**Published:** 2016-03-10

**Authors:** Katarzyna Kolasa, Krzysztof M. Zwolinski, Zoltan Kalo, Tomasz Hermanowski

**Affiliations:** Department of Health Economics, Nicolaus Copernicus University Collegium Medicum, Sandomierska 16, 85-830 Bydgoszcz, Poland; Advanced Management Training Programme in Pharmacoeconomics, Pharmaceutical Marketing and Law, Warsaw University of Technology Business School, Warsaw, Poland; Department of Health Policy & Health Economics, Faculty of Social Sciences, Eötvös Loránd University (ELTE), Budapest, Hungary

## Abstract

**Background:**

The objective of this study was to assess the potential impact of the implementation of multiple-criteria decision analysis (MCDA) on the Polish pricing and reimbursement (P&R) process with regard to orphan drugs.

**Methods:**

A four step approach was designed. Firstly, a systematic literature review was conducted to select the MCDA criteria. Secondly, a database of orphan drugs was established. Thirdly, health technology appraisals (HTA recommendations) were categorized and an MCDA appraisal was conducted. Finally, a comparison of HTA and MCDA outcomes was carried out. An MCDA outcome was considered positive if more than 50 % of the maximum number of points was reached (base case). In the sensitivity analysis, 25 % and 75 % thresholds were tested as well.

**Results:**

Out of 2242 publications, 23 full-text articles were included. The final MCDA tool consisted of ten criteria. In total, 27 distinctive drug-indication pairs regarding 21 drugs were used for the study. Six negative and 21 positive HTA recommendations were issued. In the base case, there were 19 positive MCDA outcomes. Of the 27 cases, there were 12 disagreements between the HTA and MCDA outcomes, the majority of which related to positive HTA guidance for negative MCDA outcomes. All drug-indication pairs with negative HTA recommendations were appraised positively in the MCDA framework. Economic details were available for 12 cases, of which there were 9 positive MCDA outcomes. Amongst the 12 drug-indication pairs, two were negatively appraised in the HTA process, with positive MCDA guidance, and two were appraised in the opposite direction.

**Conclusions:**

An MCDA approach may lead to different P&R outcomes compared to a standard HTA process. On the one hand, enrichment of the list of decision making criteria means further scrutiny of a given health technology and as such increases the odds of a negative P&R outcome. On the other hand, it may uncover additional values and as such increase the odds of positive P&R outcomes.

**Electronic supplementary material:**

The online version of this article (doi:10.1186/s13023-016-0388-0) contains supplementary material, which is available to authorized users.

## Background

Health technology assessment (HTA) is defined as “a multidisciplinary process that summarises information about the medical, social, economic and ethical issues related to the use of a health technology in a systematic, transparent, unbiased, robust manner. Its aim is to inform the formulation of safe, effective, health policies that are patient focused and seek to achieve best value” [1] In practice, a pricing and reimbursement (P&R) process based on HTA outcomes is very often only restricted to the review of clinical effectiveness, cost-effectiveness and budget impact. Excluding other aspects from the decision making framework meets with justifiable criticism. Having acknowledged that challenge, experts are currently pursuing new approaches that could go beyond standard HTA forms and allow for further evaluation of wider societal benefits in the decision making process.

Culyer very explicitly advocated for abandoning the current limitations of the HTA algorithm and searching for new approaches that go beyond the boundaries established by the current form of HTA analysis [2]. In the checklist that was developed, he identified 13 different ethical items to be considered by the HTA framework. Others go even further than that and suggest more advanced solutions such as multiple-criteria decision analysis (MCDA) [3]. In principle, this is a set of methods and approaches to aid decision-making, where decisions are based on multiple, often conflicting, criteria [4], and this approach has already been widely adopted in different areas of science and industry [5]. Discussion regarding its applicability to the Pricing & Reimbursement process for medicines has already been initiated as well. In addition to the development of ISPOR Task Force guidelines, the first examples of its adaptation to the healthcare decision making process have started to emerge [6].

The objective of this study was to assess the impact of the implementation of MCDA into the P&R process with regard to rare diseases. The choice of orphan drugs was based on the belief that trade-off between non-economic and economic criteria is most visible there. As far as the ethical aspects are concerned, it has to be acknowledged that 50 % of rare diseases lead to chronic disability and significantly reduce quality of life. The Committee for Orphan Medicinal Products estimated that between 2000 and 2005 as many as 40 % of health states for which orphan medicinal products had been authorized did not have any therapeutic alternative [7]. As far as the economic burden of disease is concerned, prices for orphan drugs are a particularly important challenge in the P&R decision making process [8]. This is not surprising if one takes into consideration the fact that the median price of orphan drugs has almost doubled since 2010 and was already 19 times higher than non-orphan products in 2014 [9].

Having acknowledged multiple challenges in the field of rare diseases, the EU Commission has already launched a number of projects that aim to improve patients’ access to orphan drugs [10], including the establishment of the Mechanism of Coordinated Access to Orphan Drugs working group [11]. Among its key deliverables, the Transparent Value Framework (TVF) was developed [12]. Its objective was really to construct the list of criteria contributing to the value of new orphan drugs and design a framework which helps to determine how a new medicine fulfils each of them at a given point in time. As such, the established TVF can be considered as one of the very first attempts towards the implementation of MCDA in the P&R process for orphan drugs. However, there is still limited understanding with respect to the potential impact of MCDA adaptation on the HTA outcomes. Therefore the objective of this study was set to provide relevant insight with regard to this.

Central Eastern European (CEE) settings were deliberately chosen for the purpose of this research. As conflict between economic and non-economic criteria is very feasible there, it offers a unique framework for the assessment of MCDA from both historical and contextual perspectives. The available evidence suggests that CEE societies attribute great value towards the principles of equity and moral justice [13–15]. At the same time, the scarcity of healthcare resources prove extremely challenging in making room for non-economic criteria in the decision making processes.

To our knowledge, at the present time there are no real life examples of the implementation of MCDA to the P&R decision making process regarding rare diseases in the CEE Region, and so it would be interesting to verify whether the adaptation of MCDA would improve access to orphan therapies. In particular, it is thought provoking to ask whether the implementation of an MCDA approach to the HTA process would have produced different outcomes from that of a standard HTA process.

As the biggest country in the CEE region, Poland was selected for the purpose of this study. In 2012, roughly 6,8 % of Polish GDP was spent on health care and this was one of the smallest shares amongst OECD countries [16]. In contrast to the jurisdiction of other CEE countries, HTA recommendations are made publicly available by the Polish HTA Agency (AHTAPoL) which enables such a study to be conducted. In addition to that, the choice of Poland met the expectation of some international experts who had indicated a lack of evidence regarding the P&R status of orphan drug technologies in this particular setting [17].

The Polish HTA process consists of two steps [18]. Firstly, AHTAPoL’s analytical team prepares the assessment of the HTA dossier submitted by the manufacturer. Usually, it consists of clinical effectiveness, cost effectiveness and budget impact analysis. Secondly, appraisal of the assessment report alongside other relevant documents, including the HTA dossier is conducted by the Consultative Council (the Appraisal Body). As a result, either negative or positive HTA guidance is issued. The latter can be prepared with or without restrictions and is only a recommendation. Hence the Ministry of Health is not compelled to follow the advice of the Appraisal Body.

To reach the objective of this study, a four step approach was taken. Firstly, an MCDA tool was developed. Secondly, a database of orphan drugs reviewed by the AHTAPoL (dataset) was established. Thirdly, a categorization of HTA recommendations and MCDA appraisals was conducted. Finally, a comparison of HTA recommendations and MCDA outcomes was carried out.

## Methods

### The development of and MCDA tool

Firstly a systematic literature review was conducted. Its objective was to elicit the criteria used in the decision making processes regarding both pricing and reimbursement and the implementation of orphan drug technology to clinical settings. A literature search was conducted in the PubMed Database in September 2014. The following keywords and their combinations were adopted: orphan diseases, rare diseases, neglected diseases, health technology assessment, HTA, pharmacoeconomics, reimbursement, cost effectiveness, evaluation criteria, multi criteria decision analysis, MCDA. Only full text articles written in English and concerning EU settings were included. A grey literature search was also conducted. Both research publications and non-original articles (such as summaries and reviews) were included. Conference abstracts, supplements and comments were excluded. No time limits were imposed.

In the first step, two independent reviewers evaluated each publication separately and agreed on its inclusion. Any disagreements were solved by consensus. In the second step, both reviewers studied each publication independently in order to elicit the evaluation criteria used in each particular case. A comparison of elicitation tables were produced afterwards. To avoid double counting a lack of correlation with other adopted measures was ensured. Only those which were mentioned in at least two original articles were included. In the last step, a final table was drawn up with the evaluation criteria agreed by both reviewers.

Secondly, a simple MCDA linear additive model called a “value measurement model” was constructed. It is one of the most widely used value measurements for modeling approach [19]. It is based on overall values V(a) to combine particular scores and weights brought out for selected orphan medicinal products. The overall values V(a) are usually obtained by aggregating the particular value scores received for all relevant partial criteria. The additive aggregation is formulated by the following equation as:

$$ V(a)={\displaystyle {\sum}_{i=1}^n\omega ivi} $$

Where:

V(a): overall value (OV)
ω_i_: relative importance (weight) of the ith criterion
v_i_: the performance value score (partial value functions) of the ith criterion

To simplify the assessment of orphan medicinal products under the proposed MCDA framework it was assumed that all relative weights are equal, so v_i_ for all instances has value equal to unity (v_i_). The rating scale for particular value scores (vi) ranged from 0 points at the worst outcome to 2 points at the best outcome. After the expected performance of each medicine technology was conducted against selected criteria, an aggregation of all partial scores followed to indicate the overall value (OE).

### Database of orphan drugs reviewed by the AHTAPoL

In the second step, HTA recommendations issued by the AHTAPoL agency in the period 2007–2011 concerning drug technologies with orphan indication were extracted from the Polish HTA agency website. Each indication for the same active substance was considered individually and treated as a distinctive case (drug-indication pair).

### HTA outcomes

HTA recommendations were grouped into positive and negative ones. The first ones were further divided into guidance without and with conditions. Limitations on time or the size of population were distinguished as restrictions, as well as the condition regarding the reduction of the cost of therapy. The negative HTA recommendations were grouped either by clinical or economic reasons for rejection. In the case of both of these being mentioned, the HTA recommendation was classified as negative based on clinical reason.

### MCDA outcomes

The MCDA tool that was developed was utilized to appraise each drug technology with orphan indication evaluated by AHTAPoL. In order to establish the required evidence as per each MCDA’s criteria, HTA recommendations and other relevant documents published on AHTAPoL’s website, as well as the European Public Assessment Reports (EPARs) and Scientific Discussion reports (SDRs) of the European Medicines Agency (EMEA), were utilized. Data gaps were complemented by support from published review papers [20]. Every drug-indication pair was appraised by each MCDA criteria separately and scored accordingly by two reviewers independently. Any disagreements were solved by consensus.

Given that CEA (cost effectiveness analysis) and BIA (budget impact analysis) results were not always revealed, two separate appraisals using an MCDA tool without and with economic criteria were conducted. In case CEA or BIA results were confidential or missing, a given drug-indication pair was utilized for MCDA appraisal without economic criteria only.

MCDA outcome was considered positive if more than 50 % of the maximum number of points was reached. Otherwise, a negative MCDA outcome was assumed. In addition to 50 % (base case), two other thresholds were chosen for the purpose of the sensitivity analysis, these were 25 % and 75 % of the maximum number of points respectively.

### Comparison of HTA recommendations and MCDA outcomes

In total, four different sets of comparisons were constituted, of which two had the same and two had conflicting outcomes.

## Results

### Development of an MCDA framework

**Fig. 1:**
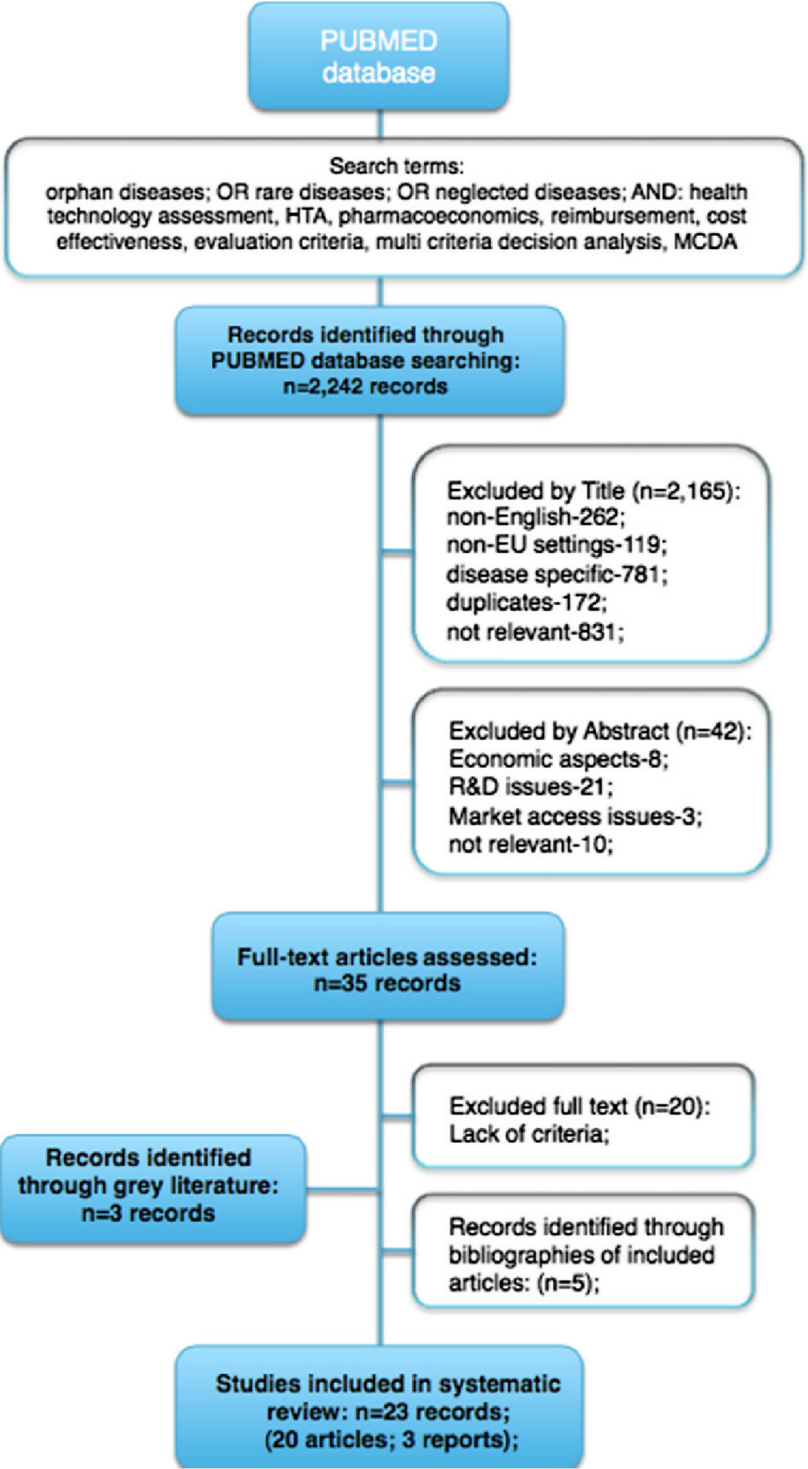
Systematic review flow. A literature search was conducted in the PubMed Database in September 2014. The following keywords and their combinations were adopted: orphan diseases, rare diseases, neglected diseases, health technology assessment, HTA, pharmacoeconomics, reimbursement, cost effectiveness, evaluation criteria, multi criteria decision analysis, MCDA. Only full text articles written in English and concerning EU settings were included. A grey literature search was also conducted.

A total of 2242 records were identified from the PubMed Database. 2165 publications were excluded following title screening. Out of the remaining 77 full-text articles, 42 did not meet the inclusion criteria. Overall 35 full-text articles were reviewed of which only 15 reported any criteria to be applied in the MCDA tool. Additionally eight publications were identified from the references of the articles initially searched. If different versions of the same documents were published, only the latest version was reviewed and included if relevant. The systematic literature review flow is presented in Fig. 1.

In the course of the systematic literature review, ten and nine criteria were selected and excluded for the MCDA tool respectively (Additional file 1: Tables S1 and S2). The key requirement for the inclusion of any MCDA criteria was to be mentioned in at least two publications. The main reason for the exclusion was the overlap with any of those already included. The key characteristics of the studies included can be found in Additional file 1: Table S3.

### Database of orphan drug-indication pairs

In total, 27 HTA guidances with regard to orphan drug technologies were issued in the period between 2007 and 2011 by AHTAPoL. In summary, 21 drug technologies were included in this study, which represents 26 % of the overall number of all known orphan drug technologies registered in the EU by the end of 2011 [21]. Out of 27 HTA recommendations, economic considerations were only revealed in 12 cases.

### HTA outcomes

Out of 27 cases related to 21 active substances, six (22 %) received negative and 21 (78 %) received positive recommendations (Table 1). Clinical reasons prevailed amongst negative HTA guidance. The majority of positive HTA recommendations were restrictive. Reimbursement limited to a specific subpopulation of patients was recommended in most occasions.

**Table 1:** HTA recommendations issued between 2007 and 2011 for orphan drug technologies by AHTAPol.

No.	Brand name (active substance)	Indication	HTA guidance	Reason for HTA recommendation
1	Fabrazyme (Agalsidase beta)	Fabry disease (alpha-galactosidase A deficiency) – long-term replace therapy	Negative	Insufficient clinical effectiveness, safety concerns, not cost-effective vs standard of care
2	Elaprase (Idursulfase)	Mucopolysaccharidosis type II, MPS II (Hunter syndrome) – long-term treatment	Negative	Insufficient clinical effectiveness
3	Kuvan (Tetrahydrobiopterin)	Hyperphenylalaninemia (HPA) in patients with tetrahydrobiopterin (BH4) deficiency	Positive	Restricted with time limits
4	Increlex (Mecasermin)	Insulin-like growth factor deficiency -IGFD (Laron Syndrome) – long-term treatment	Positive	Restricted with time limits
5	Somavert (Pegvisomant)	Acromegaly	Negative	Insufficient clinical effectiveness, not cost-effective vs standard of care
6	Ventavis (Iloprost)	Pulmonary arterial hypertension (PAH)	Positive	Restricted to subpopulations
7	Tracleer (Bosentan)	Pulmonary arterial hypertension (PAH)	Positive	Restricted to subpopulations
8	Cystadane (Betaine anhydrous)	Homocystinuria	Positive	Unrestricted
9	Zavesca (Miglustat)	Niemann-Pick type C syndrome (disease)	Positive	Restricted with time limits, conditional on the reduction of cost of therapy
10	Volibris (Ambrisentan)	Pulmonary arterial hypertension (PAH)	Positive	Unrestricted
11	Nexavar (Sorafenib)	Renal cell carcinoma (RCC)	Negative (2008)	Insufficient clinical effectiveness, not cost-effective vs standard of care, unacceptable budget impact
12	Nexavar (Sorafenib)	Renal cell carcinoma (RCC)	Negative (2009)	Not cost-effective vs standard of care, unacceptable budget impact
13	Nexavar (Sorafenib)	Hepatocellular carcinoma (HCC)	Positive	Restricted to subpopulations
14	Nplate (Romiplostim)	Chronic immune (idiopathic) thrombocytopenic purpura (ITP)	Positive	Restricted to subpopulations
15	Torisel (Temsirolimus)	Renal cell carcinoma (RCC)	Negative	Insufficient clinical effectiveness, not cost-effective vs standard of care
16	Tasigna (Nilotinib)	Chronic myeloid leukemia (CML)	Positive	Restricted to subpopulations, conditional on the reduction of cost of therapy
17	Vidaza (Azacitidine)	Acute myelogenous leukemia (AML)	Positive	Unrestricted
18	Glivec (imatinib)	Philadelphia chromosome positive chronic myeloid leukemia (ALL Ph+)	Positive	Unrestricted
19	Glivec (imatinib)	Myelodysplastic/myeloproliferative diseases (MDS/MPD)	Positive	Unrestricted
20	Glivec (imatinib)	Dermatofibrosarcoma protuberans (DFSP)	Positive	Unrestricted
21	Glivec (imatinib)	Malignant gastrointestinal stromal tumors (GIST)	Positive	Unrestricted
22	Revlimid (Lenalidomide)	Multiple myeloma (MM)	Positive	Restricted to subpopulations
23	Revlimid (Lenalidomide)	Myelodysplastic/Myeloproliferative syndrome (MM/S) (off-label indication)	Positive	Off-label indication, restricted to subpopulations, conditional on the reduction of cost of therapy
24	Yondelis (Trabectedin)	Soft tissue sarcoma	Positive	Restricted to subpopulations, conditional on the reduction of cost of therapy
25	Sprycel (Dasatinib)	Chronic myeloid leukemia (CML)	Positive	Restricted to subpopulations
26	Revatio (Sildenafil)	Pulmonary arterial hypertension (PAH)	Positive	Unrestricted
27	Atriance (Nelarabine)	T-cell acute lymphoblastic leukemia	Positive	Restricted to subpopulations, conditional on the reduction of cost of therapy

### MCDA outcomes

The MCDA framework that was developed is presented in Table 2. In the base case, there were 19 (70 %) and nine (75 %) positive MCDA outcomes respectively without and with economic criteria being included (Tables 3 and 4).

**Table 2:** The list of MCDA criteria

No.	Main criterion	Partial criteria together with corresponding weights
1.	Indication uniqueness	a) one unique indication (2 points), b) more than one orphan indications (1 point), c) one or more indications for common disease (0 points)
2.	Disease rarity	a) prevalence < 0,5 per 10,000 UE citizens (2 points), b) prevalence in the range of 0,5 and 1 per 10,000 UE citizens (1 point), c) prevalence >1 per 10,000 UE citizens (0 points)
3.	Disease severity	a) high mortality often with poor prognosis e.g. cancers (2 points) b) chronic without high mortality and morbidity (1 point), c) severe invalidity, severely harm of capacities central to individuals’ functioning in society – e.g. hearing, eyesight etc. (1 point)
4.	Advancement of technology	a) Advanced therapy medicinal product (ATMP) including biopharmaceutical, innovative synthetic entities and delivery systems as well non-biological complex drug (2 points), b) conventional small molecule (molecular weight, MW < 500 Da) with at least one stereogenic (chiral) center in its structure (1 point), c) widely available simple chemical entities (e.g. zinc acetate) including molecules easily synthesized from commercially available precursors - fine chemicals – (0 points)
5.	Manufacturing technology complexity	a) expensive biotechnological processes (2 points) b) complex synthetic path consisting of at least three independent chemical transformations (1 point) c) manufacturing require the use of separation techniques for most intermediates (1 point)
6.	Therapeutic alternative (unmet medical need)	a) no comparable alternative available (2 points) b) second line treatment available (1 point) c) at least one comparable alternative available (0 points)
7.	Scientific evidence for clinical efficiency (level of uncertainty)	a) randomised placebo(or active)-controlled clinical trial(s) (RCT) with hard endpoints such as overall survival or time to progression (TTP) (2 points), b) randomised placebo(or active)-controlled clinical trial(s) (RCT) with surrogate endpoints (1 points), c) uncontrolled non-randomised clinical trial(s), observation studies or cohort studies with relevant but only limited level of uncertainty (1 point), d) very limited data with high level of uncertainty – e.g. case reports (0 points)
8.	Benefits from use of medicine (safety and adverse effects)	a) Only minor and reversible adverse events (2 points) b) Low incidence of severe of adverse events (1 point) c) High incidence of severe of adverse events (0 point)
9.	Cost effectiveness	a) ICER below 24 k€ (2 points) b) ICER in the range between 24 k€ and 48 k€ (1 point) c) ICER above 48 k€ (0 points)
10.	Budget impact (in €)	Total costs of reimbursement in first two years: a) budget savings or positive budget impact below 1,2 M€ (2 points) b) in the range between 1,2 and 2,4 M€ (1 point) c) above 2,4 M€ (0 points)

**Table 3:** MCDA outcomes for drug-indication pairs appraised by AHTAPol between 2007 and 2011 (economic criteria excluded)

No.	Drug-indication pair	Indication uniqueness	Disease rarity	Disease severity	Adv.tech.	Manufacturing technology	Therapeutic alternative	Sci. evid. clin.eff.	Benefits from use of medicine	Total number of points
1	Elaprase	2	2	1	2	2	2	1	1	13
2	Fabrazyme	2	2	2	2	2	0	1	1	12
	THRESHOLD	75 %								12 points
3	Nexavar (HCC)	1	2	2	0	2	2	2	0	11
4	Kuvan	0	2	1	1	2	2	1	2	11
5	Nplate	2	1	0	2	2	2	1	1	11
6	Cystadane	2	2	2	0	0	2	0	2	10
7	Yondelis	1	1	2	2	0	2	2	0	10
8	Somavert	2	1	1	2	2	0	1	1	10
9	Tasigna	2	2	2	0	0	2	1	1	10
10	Increlex	2	0	0	2	2	2	1	1	10
11	Glivec – (GIST)	0	2	2	0	1	2	2	0	9
12	Revlimid (MM/S)	1	0	2	1	0	2	2	1	9
13	Glivec (DFSP)	0	2	2	0	1	2	1	1	9
14	Torisel	1	0	2	2	2	0	1	1	9
15	Ventavis	2	0	1	1	2	0	1	1	8
16	Zavesca	1	1	2	1	0	0	1	2	8
17	Tracleer	1	1	1	0	2	0	1	2	8
18	Volibris	2	2	0	1	0	0	1	2	8
19/20	Nexavar (RCC)	1	0	2	0	2	1	2	0	8
	THRESHOLD	50 %								8 points
21	Vidaza	1	0	2	1	0	0	2	1	7
22	Sprycel	1	1	2	0	0	1	1	1	7
23	Revlimid (MM)	1	0	2	1	0	0	2	1	7
24	Atriance	1	0	2	1	0	2	1	0	7
25	Glivec (ALL Ph+)	0	1	2	0	1	0	1	0	5
26	Revatio	0	1	1	0	0	0	1	2	5
27	Glivec (MDS/MPD)	0	0	2	0	1	0	1	0	4
	THRESHOLD	25 %								4 points

*Adv.tech.* Advancement of technology, *Sci. evid. clin.eff.* scientific evidence for clinical efficiency, *HCC* Hepatocellular carcinoma, *GIST* Malignant gastrointestinal stromal tumors, *MM/S* Myeloproliferative syndrome, *DFSP* Dermatofibrosarcoma protuberans, *RCC* Renal cell carcinoma, *MM* Multiple myeloma, *ALL Ph+* Philadelphia chromosome positive chronic myeloid leukemia, *MDS/MPD* Myelodysplastic/myeloproliferative diseases

**Table 4:** MCDA outcomes for drug-indication pairs appraised by AHTAPol between 2007 and 2011 (economic criteria included)

No.	Drug-indication pair	Indication uniqueness	Disease rarity	Disease severity	Adv.tech.	Manufacturing technology	Therapeutic alternative	Sci. evid. clin.eff	Benefits from use of medicine	Cost effectiveness	Budget impact	Total number of points
	THRESHOLD	75 %										15 points
1	Nexavar (HCC)	1	2	2	0	2	2	2	0	1	1	13
2	Nplate	2	1	0	2	2	2	1	1	1	1	13
3	Cystadane	2	2	2	0	0	2	0	2	0	2	12
4	Increlex	2	0	0	2	2	2	1	1	0	2	12
5	Revlimid (MM/S)	1	0	2	1	0	2	2	1	1	2	12
6	Yondelis	1	1	2	2	0	2	2	0	1	0	11
7	Somavert	2	1	1	2	2	0	1	1	1	0	11
8	Tasigna	2	2	2	0	0	2	1	1	0	0	10
9	Torisel	1	0	2	2	2	0	1	1	0	1	10
	THRESHOLD	50 %										10 points
10	Vidaza	1	0	2	1	0	0	2	1	1	0	8
11	Atriance	1	0	2	1	0	2	1	0	0	1	8
12	Nexavar (RCC)	1	0	2	0	2	1	2	0	0	0	8
	THRESHOLD	25 %										5 points

*Adv. tech.* Advancement of technology, *Sci. evid. clin. eff.* scientific evidence for clinical efficiency, *NA* no data available, *HCC* Hepatocellular carcinoma, *GIST* Malignant gastrointestinal stromal tumors, *MM/S* Myeloproliferative syndrome, *DFSP* Dermatofibrosarcoma protuberans, *RCC* Renal cell carcinoma, *MM* Multiple myeloma, *ALL Ph+* Philadelphia chromosome positive chronic myeloid leukemia, *MDS/MPD* Myelodysplastic/myeloproliferative diseases

In total, the majority of points were granted to the severity of the disease in question and the evidence of clinical effectiveness. At the same time, the lowest scores were assigned to the availability of alternative technology and economic criteria if available.

In the group of drug-indication pairs above the 50 % threshold, indication uniqueness and lack of therapeutic alternative were scored highest. In the group of cases below the 50 % threshold, advancement of technology and the results of CEA and BIA, if available, received the lowest number of points.

Irrespective of whether the ranking included or excluded economic criteria, Elaprase and Fabrazyme received the highest and Glivec (MDS/MPD) the lowest number of points.

### Comparison of HTA and MCDA outcomes

#### Agreement between HTA and MCDA outcomes

There were 12 (44 %) cases of agreement between HTA and MCDA if economic criteria were excluded in the base case (Tables 5 and 6). The corresponding number was 8 (66 %) if economic criteria were included. The majority of agreements related to positive outcomes. There was only one example of consistency with respect to negative recommendations. This happened when economic criteria were taken into consideration.

**Table 5:** A comparison of HTA and MCDA outcomes (economic criteria excluded) for 50 % threshold, multiple HTA restrictions imposed

		HTA					
		Positive				Negative	
		Unrestricted	Time restrictions	Limits to specific subpopulation	Finanacial restrictions	Clinical reasons	Economic reasons
MCDA	Positive	Cystadane, Volibris, Torisel, Glivec (DFSP), Glivec (GIST)	Zavesca, Kuvan, Increlex	Ventavis, Tracleer, Nexavar (HCC), Nplate, Tasigna, Glivec (MM), Yondelis	Zavesca, Tasigna, Yondelis	Elaprase, Fabrazyme, Somavert, Torisel	Fabrazyme, Somavert, Torisel, Nexavar (RCC)
	Negative	Vidaza, Glivec (ALL Ph+), Glivec (MDS/MPD), Revatio	None	Revlimid (MM/S), Sprycel, Atriance)	Revlimid (MM/S), Atriance	None	None

**Table 6:** A comparison of HTA and MCDA outcomes (economic criteria included) for 50 % threshold, multiple HTA restrictions imposed

		HTA					
		Positive				Negative	
		Unrestricted	Time restrictions	Limits to specific subpopulation	Finanacial restrictions	Clinical reasons	Economic reasons
MCDA	Positive	Cystadane, Volibris, Torisel	Kuvan, Increlex	Nexavar (HCC), Nplate, Tasigna, Glivec (MM), Yondelis	Tasigna, Yondelis	Elaprase, Fabrazyme, Somavert, Torisel	Fabrazyme, Somavert, Torisel
	Negative	Vidaza, Glivec (ALL Ph+), Glivec (MDS/MPD), Revatio, Glivec (DFSP), Glivec (GIST)	Zavesca	Tracleer, Ventavis, Sprycel, Atriance, Revlimid (MM/S)	Zavesca, Atriance, Revlimid (MM/S)	None	Nexavar (RCC)

#### Disagreement between HTA and MCDA outcomes

##### Positive HTA vs negative MCDA outcome

In the base case there were seven and two cases of negative MCDA outcomes for positive HTA recommendations respectively without and with economic criteria being included (Tables 5 and 6). Amongst these disagreements, AHTAPoL’s unrestricted guidances prevailed and were followed by restrictions to subpopulation in both groups. At the same time, the lowest MCDA scores were given to the criteria of availability of alternative treatment options, complexity of manufacturing technology, disease rarity and advancement of technology.

#### Negative HTA vs positive MCDA outcome

In the base case, there were five and two cases respectively of negative HTA recommendation appraised positively with the MCDA tool without and with economic criteria being included. While insufficient clinical evidence was raised as a key reason for a negative HTA recommendation, it was indication uniqueness, disease severity and rarity which were scored highest in the MCDA appraisal process.

### Sensitivity analysis

25 % and 75 % thresholds were applied for MCDA appraisal in the sensitivity analysis. If economic criteria were excluded, there would only have been two drug-indication pairs with a positive MCDA outcome for the 75 % threshold. Both of them received a negative HTA recommendation. There would have only been negative MCDA outcomes if economic criteria were being included for the 75 % threshold. At the same time all cases would have received a positive MCDA appraisal if the 25 % threshold was applied irrespective of whether CEA and BIA results were to be included or excluded.

## Discussion

The objective of this study was to address the question of whether MCDA implementation in the HTA process would have changed HTA outcomes and consequently influenced the pricing and reimbursement decision making process regarding orphan therapies in Polish settings. In total, 27 individual drug-indication pairs were reviewed and evaluated through an MCDA framework.

To our knowledge this was one of very few attempts to adapt an MCDA approach to the assessment of orphan drug technologies worldwide and the first one in a CEE setting. Several authors have advocated for the application of a multi-criteria decision analysis framework to health technologies for rare diseases but so far only limited research has been published. Hence our study with a sample of as many as 27 individual cases can be clearly distinguished from other pilot studies [22].

This research provides some interesting insights into why the adaptation of an MCDA approach may lead to different P&R outcomes compared to a standard HTA process. On the one hand, the enrichment of the list of decision making criteria means further scrutiny of the health technology in question and as such increases the chances of negative P&R outcomes. On the other hand the inclusion of wider societal perspectives may uncover additional values of a given health technology and as such increase the chances of positive P&R outcomes.

As far as the broadening of a P&R decision making basis is concerned, the study revealed that there were more negative MCDA outcomes for positive HTA recommendations than the other way round. While MCDA ratings were consistent with HTA outcomes regarding the criteria used in processes such as clinical effectiveness and safety considerations, it was the other aspects that contributed most to a negative MCDA appraisal. An interesting example of how additional criteria may influence the P&R process could be the notion of the availability of alternative therapy being introduced into the MCDA tool. While the majority of cases above the 50 % threshold related to orphan drugs without a comparator, most drug-indication pairs scored below the threshold concerned for rare conditions for which an available treatment option exists. In the latter case, the exception was Atriance, which was positively appraised by AHTAPoL but gained a negative MCDA outcome. In that particular case, the claim of a missing comparator is questionable if one takes into consideration ‘trials of various chemotherapeutic regimens based on individual responses’ which may indicate the existence of some alternative treatment options [23].

Looking into the potential impact of MCDA’s implementation into the P&R process, it is interesting to note that the economic considerations tended to play a less important role in MCDA appraisal compared to the HTA process. An example in case could be Elapraze and Fabrazyme. Both drugs were appraised negatively by AHTAPoL but were scored the highest in the MCDA appraisal process. On the one hand, the lack of a positive HTA recommendation is not surprising if one takes into account the fact that both products are positioned amongst the world’s most expensive drugs [24]. On the other hand, the multi criteria approach allowed economic arguments against other characteristics of the drug technologies in question to be balanced. Unless no differentiation across weights is applied, the adaptation of a holistic approach may produce another hurdle for payers concerned with budget impact. In this context it could be mentioned that the economic considerations did not influence the result of the MCDA appraisal to any significant extent. With the 50 % threshold scenario, there were only two changes of MCDA outcomes after the economic criteria were included. While MCDA was revised from negative to positive for Revlimid (MM), the opposite occurred for Nexavar (RCC). At the same time, affordability concerns were mentioned in as many as 66 % of negative HTA guidances.

Our study is not limitation free. Firstly, it has to be acknowledged that subjectivity of the adaptation of the MCDA tool might have introduced some bias into the results. As far as the selection of criteria is concerned, we deliberately excluded some of them to avoid ambiguity. One exception was with respect to the ‘advancement of technology’, which defines a product’s innovation in terms of its active ingredients. The reason for its inclusion is that it indirectly correlates with the level of investment in R&D (research and development) and manufacturing requirements [25]. As far as the appraisal process is concerned, MCDA outcomes are based on the subjective judgment of only two reviewers which could have introduced some additional bias to the results. The incorporation of more experts’ opinions could have further improved the external validity of the MCDA process. It certainly has to be taken into consideration prior to any generalization of the results of our study.

Secondly, although some authors emphasize the important limitation of monetary criteria such as cost-effectiveness analysis, evaluation of economic data was also performed [26]. This was not without challenges as the full transparency of HTA reports published on the AHTAPoL website was missing. This is especially true with regard to the details of cost effectiveness and budget impact analysis. In the majority of cases where economic data were revealed, it was actually unfavorable budget impact results that impacted MCDA to the greatest extent. The appraisal might have led to different outcomes if CEA and BIA results were available.

Finally, arbitrariness in the implementation of an MCDA approach such as the choice of MCDA methods and the lack of weighting of adopted criteria could have biased the results as well. We chose a simple linear additive model because of its simplicity. It was believed to be consistent with the way people usually made aggregation as well. We do however have to acknowledge that there are a number of drawbacks that might have impacted on our results. As far as weights are concerned, we adopted a very simple approach with a range from 0 to 2 points per criterion irrespective of its importance. There is some reason to believe that the process of assigning weights should be based on discrete choice experiments with the involvement of the representatives of society, experts in the areas of health, as well as economists, and finally patients and their caregivers [22]. Such weights are very likely to be jurisdiction specific [22, 27]. For example Mentzakis et al. performed discrete choice experiments on a convenience sample of university students in Canada to investigate individual preferences regarding public funding for rare and common diseases [28]. Surprisingly they found that the respondents did not have a preference for the government to spend more for drugs used to treat rare diseases.

Our contention is that an MCDA approach allows the decision-making process to be structured under conditions of better transparency and to support efforts for more equitable distribution of healthcare public funds. Even though our study does not provide evidence that multi criteria analysis leads to a less restrictive decision making process, we are cautious that the scarcity of financial resources might trigger some resistance among decision makers regarding the implementation of an MCDA approach to the P&R processes for orphan drugs in the CEE Region. The good news is that National Health Fund spending for rare diseases has increased by almost threefold in Poland since 2009 [29]. As long as rare diseases are funded from the same budget as non-orphan conditions, the decision makers may remain unwilling to adopt different P&R rules for their evaluation. Experiences from more developed countries indicate that separate financing mechanisms may exist. For example, a special fund called ‘AIFA 5 %’ was established in Italy [30]. In England and Wales, drug technologies for very rare diseases are currently assessed and commissioned by NICE distinctly from other medicines used to treat more common diseases [31]. In Scotland a special fund dedicated to orphan medicinal products was set up in 2013 as well [32]. In Australia some parts of very expensive drug technologies are reimbursed under a special Life Saving Drug Program (LSDP) [33]. Drug technologies qualify for LSDP if they are clinically necessary and effective, and cannot meet the cost-effectiveness criteria [33].

However, a separate financing mechanism does not seem to be the only pre requisite condition for the successful implementation of an MCDA approach. Awareness of the need to adapt a wide range of P&R rules with respect to the evaluation of orphan drugs has to be raised as well. Input from experts is therefore required to encourage dialogue between the general public and the decision makers. Given the variation of social values across jurisdictions, the list of criteria and their relative weights requires incorporation of country specific preferences. Hence it is unlikely that one standardized pan-European set of rules could be successful. The efforts of experts should therefore rather focus on the development of a country specific MCDA framework instead. Nevertheless an EU patient registry project could be of great value in the pursuit of more equitable distribution of healthcare resources for rare diseases as well. Not only could such an endeavor help in collecting real life data but also in handling uncertainty regarding clinical outcomes. It could be enormously beneficial to both decision makers and patients when making their national and individual decisions regarding treatment choices.

## Conclusions

As our study revealed, an MCDA approach may lead to different P&R outcomes compared to a standard HTA process. On the one hand, enrichment of the list of decision making criteria leads to further scrutiny of the given health technology and as such may increase the odds of a negative P&R outcome. On the other hand, it uncovers additional values and as such may increase the odds of positive P&R outcomes.

In competition with common disease, where the incremental gains are more significant, the pricing and reimbursement decision making process for the treatment of rare diseases will remain challenging. There is a growing understanding that the allocation criteria currently adopted, such as the cost effectiveness threshold, does not allow the value of orphan drugs to be fully captured [34]. It is therefore hoped that our study could contribute to the discussion on the overall appropriateness of the adaptation of an MCDA approach in CEE settings and its usefulness in the search for more transparent and equitable resource allocation in the healthcare sector.

## Additional file

Additional file 1: Table S1. Included criteria in MCDA framework, [22, 26, 28, 34, 35, 37–44, 46–53]. Table S2. Excluded criteria from MCDA framework [22, 26, 28, 34–36, 38–45, 47, 48, 50, 51]. Table S3. Key characteristics of included studies. (DOCX 491 kb)
