# Supplementary material for: Potential impact of the implementation of multiple-criteria decision analysis (MCDA) on the Polish pricing and reimbursement process of orphan drugs
Source: Orphanet J Rare Dis. 2016 Mar 10;11:23. doi: 10.1186/s13023-016-0388-0 (PMC4787054; doi:10.1186/s13023-016-0388-0)
Supplement: Additional file 1: Table S1. — Included criteria in MCDA framework, [22, 26, 28, 34, 35, 37–44, 46–53]. Table S2. Excluded criteria from MCDA framework [22, 26, 28, 34–36, 38–45, 47, 48, 50, 51]. Table S3. Key characteristics of included studies. (DOCX 491 kb) [file 13023_2016_388_MOESM1_ESM.docx]

Supplementary Table 1 - Included criteria in MCDA framework.

| No. | Criteria | Definition | Synonymous | Data resources (references) |
| --- | --- | --- | --- | --- |
| 1 | Indication uniqueness | the number of approved distinctive indications of active substance |  | [34], [44], |
| 2 | Therapeutic alternative | Availability of alternative treatment options for given target group | Unmet medical need, | [22], [28], [34], [38] [39], [42], [43], [47], [52] |
| 3 | Availability and quality of scientific evidence for clinical effectiveness | The credibility and robustness of clinical evidence | Effectiveness, level of uncertainty of effectiveness, evidence of health gain | [22], [28], [34], [35], [42], [43], [44], [47], [48], [49], [52], [53] |
| 4 | Disease rarity | Prevalence of the condition | Disease frequency | [28], [34], [35], [37], [49], [50], [52], [53] |
| 5 | Disease severity | The disease prognosis without treatment | disease morbidity and/or disease survival prognosis and patient clinical disability with current standard of care, impact of the disease on a pateient`s quality of life, | [28], [34], [35], [38 after 39], [40 after 41], [42] [43], [47], [48], [49], [53] |
| 6 | Advancement of technology | Treatment innovation, definied as the scientific advance of the new treatment together with contribution to patient outcome | Product innovation in terms of active ingredients. | [22], [42] |
| 7 | Manufacturing technology complexity | Manufacturing process requirements |  | [26], [34] |
| 8 | Benefits from use of medicine (Safety and adverse effects) | the impact of drug treatment on a patient’s health and comfort as a result of reducing adverse events (safety profile) | Therapeutic advantage on safety profile, improving quality of life, related with health gain and patient comfort (ease of use) | [22], [28], [37], [42], [44], [47], [48], [52], [53] |
| 9 | Budget impact | The impact on public payer budget | Budget impact is based on the expected number of patients and is linearly dependent on disease prevalence | [28], [38] [43], [47], [49], [50], [53] |
| 10 | Cost-effectiveness | ICER | Incremental cost-effectiveness ratio (ICER) | [26], [28], [35], [37], [38], [39], [40], [44], [46], [47], [48], [50], [51], [53] |

Supplementary Table 2. Excluded criteria from MCDA framework.

|  | Criteria | Definition | Reason for exclusion | Data resources (references) |
| --- | --- | --- | --- | --- |
| 1 | Rule of rescue | Imperative feel to rescue identifiable individuals facing avoidable death, notwithstanding the potentially high opportunity cost of doing so; | Rule of rescue has not yet achieved a consistent meaning. | [28], [38], [39], [45], [48], [50] |
| 2. | Innovation of medicine (value of innovation) | Potential health gain provide meaningful benefit beyond available treatments but also e.g. a new active substance which was not previously authorized in the EU Community, or new substance have unique mechanism of action | Its scope is open to interpretation which could introduce ambiguity to the assessment process – specific, narrow scope of innovation must be clarified. Overlap with indication uniqueness. | [35], [38] |
| 3. | Valuation of health gain | impact on condition / disease modification - improvements in health outcome.  Priority for treatment with potential to preserve life and if only possible completely reverse the disability rather than slow further disease progression | Overlap with benefits from use of medicine and disease severity (safety profil) | [34], [35], [43], [51] |
| 4. | Follow up measures (additional benefits and associated cost) | Additional studies designed to answer specific, defined and delineated question | Overlap with availability and quality of scientific evidence for clinical efficiency. | [34] |
| 5. | Level of research undertaken | Knowledge available at the time of starting research and development programme | Overlap with the advancement of technology | [34], [44] |
| 6. | Cost of production | Cost of developing a drug (investments in research and innovation) | Overlap with the advancement of technology. | [35] |
|  |  |  |  |  |
|  |  |  |  |  |
| 7. | Non-direct cost | Social impact of the disease on | Overlap with disease severity | [22, 42], [43] |
|  |  | patients` and carers` daily lives | and availability and quality of scientific evidence for clinical efficiency. |  |
| 8. | Level of return | Total revenues in the context of multiple indications for the same molecule owned by the same company | Hard to estimate because data are often confidential. | [42] |
| 9. | Other attributes/criteria not-classified elsewhere | Impact of the new technology, cost to the NHS and Personal Social Services, value for money, impact of the technology beyond distinct health benefits, impact of the technology on the delivery of the specialized service.  The cost to patient if the drug is not listed for public reimbursement. Extending longevity. Economic evaluation of additional benefits. Equity. Social, ethical and legal implications. Potential of the technology to prevent mortality/morbidity. Disease survival prognosis with current standard of care. ‘Societal value’. | | [22], [26], [36], [38], [39], [40], [41], [42], [47], [48] |

Supplementary Table 3. Key characteristics of included studies.

| No |  | Nature of the study [reference in the main text article] | Identified criteria |
| --- | --- | --- | --- |
| 1 | Hughes-Wilson W, Palma A, Schuurman A, Simoens S. Paying for the Orphan drug System: break or bend? Is it time for a new evaluation system for payers in Europe to take account of new rare disease treatments? Orphanet J Rare Dis. 2012; 7:74. | Original research [34] | Indication uniqueness, therapeutic alternative, availability and quality of scientific evidence for clinical effectiveness, disease rarity, disease severity, manufacturing technology complexity, valuation of health gain, follow up measures (additional benefits and associated cost), level of research undertaken. |
| 2 | Simoens S. Pricing and reimbursement of orphan drugs: the need for more transparency. Orphanet J Rare Dis 2011; 6:42. | Literature review, commentary [44] | Indication uniqueness, availability and quality of scientific evidence for clinical effectiveness, benefits from use of medicine (Safety and adverse effects), cost-effectiveness, level of research undertaken. |
| 3 | M Mentzakis, E., Stefanowska, P., Hurley, J. A discrete choice experiment investigating preferences for funding drugs used to treat orphan diseases: an exploratory study. Health  Econ Policy Law 2011; 6:405–433. | Original research, pilot study, method: discrete choice experiment[28] | Therapeutic alternative, availability and quality of scientific evidence for clinical effectiveness, disease rarity, disease severity, benefits from use of medicine (Safety and adverse effects), budget impact, cost-effectiveness, rule of rescue. |
| 4 | Drummond MF, Wilson DA, Kanavos P, et al. Assessing the economic challenges posed by orphan drugs. Int J Technol Assess Health Care 2007; 23(1):36-42. | Commentary, discussion, general essay [38] | Therapeutic alternative, disease severity, budget impact, Cost-effectiveness, rule of rescue, innovation of medicine (value of innovation), other attributes/criteria not-classified elsewhere (see Supplementary table 2). |
| 5 | George B, Harris A, Mitchell A. Cost-effectiveness analysis and the consistency of decision-making: evidences from pharmaceutical reimbursement in Australia (1991 to 1996). Pharmacoeconomics 2001; 19:1103-1109. | Original research article, methods: submissions review, comparision [39] | Therapeutic alternative, disease severity, Cost-effectiveness, rule of rescue, other attributes/criteria not-classified elsewhere (see Supplementary table 2). |
| 6 | Sussex J, Rollet P, M. Garau M, et al. Multi-criteria decision analysis to value orphan medicines. Office of Health Economics, London, 2013. | Original research, methods: literature searches and three workshops [42] | Therapeutic alternative, availability and quality of scientific evidence for clinical effectiveness, disease severity, advancement of technology, benefits from use of medicine (Safety and adverse effects), non-direct cost, level of return, other attributes/criteria not-classified elsewhere (see Supplementary table 2). |
| 7 | Sussex J, Rollet P, Garau M, et al. A pilot study of multicriteria decision analysis for valuing orphan medicines. Value Health 2013; 16:1163-1169. | Original research, pilot study, brief report [22] | Therapeutic alternative, availability and quality of scientific evidence for clinical effectiveness, advancement of technology, benefits from use of medicine (Safety and adverse effects), non-direct cost, other attributes/criteria not-classified elsewhere (see Supplementary table 2). |
| 8 | Iskrov GG, Raycheva RD, Stefanov RS. Insight into reimbursement decision-making criteria in Bulgaria: implications for orphan drugs. Folia Med (Plovdiv) 2013; 55(3-4):80- 86. | Original research, national level restriction (Bulgaria), method: critical analysis [47] | Therapeutic alternative, availability and quality of scientific evidence for clinical effectiveness, disease severity, benefits from use of medicine (Safety and adverse effects), budget impact, Cost-effectiveness, other attributes/criteria not-classified elsewhere (see Supplementary table 2). |
| 9 | Stolk P, Willemen MJ, Leufkens HG. Rare essentials: drugs for rare diseases as essential medicines. Bull World Health Organ. 2006 Sep;84(9):745-51. | Commentary [52] | Therapeutic alternative, availability and quality of scientific evidence for clinical effectiveness, disease rarity, benefits from use of medicine (Safety and adverse effects). |
| 10 | McCabe C, Claxton K, Tsuchiya A. Orphan drugs and the NHS: should we value rarity? BMJ 2005; 331(7523):1016-1019. | Commentary, Education and debate [29] | Availability and quality of scientific evidence for clinical effectiveness, disease rarity, disease severity, cost-effectiveness, innovation of medicine (value of innovation), valuation of health gain, cost of production. |
| 11 | Rosenberg-Yunger ZRS, Daar AS, Thorsteinsdottir H, Martin DK. Priority setting for orphan drugs: an international comparision. Health Policy 2011; 100:25-34. | Original research, method: qualitative case studies [48] | Availability and quality of scientific evidence for clinical, disease severity, benefits from use of medicine (Safety and adverse effects), cost- effectiveness, rule of rescue, other attributes/criteria not-classified elsewhere (see Supplementary table 2). |
| 12 | Winquist E, Coyle D, Clarke JTR, et al. Application of a policy framework for the public funding of drugs for rare diseases. J Gen Intern Med 2014; 29(Suppl 3):S774-779. | Original research, method: retrospective observational cohort study [49] | Availability and quality of scientific evidence for clinical effectiveness, disease rarity, disease severity, budget impact, |
| 13 | D. Hughes, Rationing of drugs for rare diseases, Pharmacoeconomics, 2006; 24 (4): 315-316. | Commentary [50] | Disease rarity, budget impact, cost-effectiveness, rule of rescue, other attributes/criteria not-classified elsewhere (see Supplementary table 2). |
| 14 | Hughes DA, Tunnage B, Yeo ST. Drugs for exceptionally rare diseases: do they deserve special status for funding? QJM. 2005 Nov;98(11):829-36. | Commentary [53] | Availability and quality of scientific evidence for clinical effectiveness, disease rarity, disease severity, benefits from use of medicine (Safety and adverse effects), budget impact, cost-effectiveness, other attributes/criteria not-classified elsewhere (see Supplementary table 2). |
| 15 | Hyry HI, Stern AD, Cox TM, Roos JCP. Limits on use of health economic assessments for rare diseases. Q J Med 2014; 107(3):241-245. | Commentary [26] | Manufacturing technology complexity, cost- effectiveness, other attributes/criteria not-classified elsewhere (see Supplementary table 2). |
| 16 | Simoens S, Picavet E, Dooms M, et al. Cost-Effectiveness Assessment of Orphan Drugs. Appl Health Econ Health Policy 2013; 11:1-3. | Commentary, a scientific and political conundrum [46] | Cost- effectiveness. |
| 17 | Drummond MF. Challenges in the economic evaluation of orphan drugs. Pharmaceutical policy. Eurohealth 2007; 14(2):16-17. | Commentary [37] | Disease rarity, benefits from use of medicine (Safety and adverse effects), cost- effectiveness. |
| 18 | Simoens S. Health technologies for rare diseases: does conventional HTA still apply? Expert Rev Pharmacoecon Outcomes Res 2014; 14(3):315-317. | Expert review [34] | Disease severity, cost-effectiveness, other attributes/criteria not-classified elsewhere (see Supplementary table 2). |
| 19 | Largent EA, Pearson SD. Which orphans will find a home? The rule of rescue in resource allocation for rare diseases. Hastings Cent Rep 2012; 42(1):27-34 | Commentary [39a] | Rule of rescue. |
| 20 | Sheehan M. Orphan drugs and the NHS: fairness in health care entails more than cost effectiveness. BMJ. 2005 Nov 12;331(7525):1144-5. | Commentary, letter[45] | Cost- effectiveness, valuation of health gain, other attributes/criteria not-classified elsewhere (see Supplementary table 2). |
| 21 | National Institute for Health and Clinical Excellence (NICE). Appraising Orphan Drugs, DRAFT v3. | Report [30] | Other attributes/criteria not-classified elsewhere (see Supplementary table 2). |
| 22 | National Institute for Health and Clinical Excellence NICE Citizens council report – report 4: Ultra orphan drugs. London, November 2004. | Report [37] | Therapeutic alternative, availability and quality of scientific evidence for clinical effectiveness, disease severity, budget impact, valuation of health gain, non-direct cost. |
| 23 | National Institute for Health and Care Excellence. Interim, process and methods of the highly specialized technologies programme, 2013. | Report [35] | Disease severity, other attributes/criteria not-classified elsewhere (see Supplementary table 2). |
